# What do medical students think are characteristics of a good ultrasound tutor? A qualitative study

**DOI:** 10.1186/s12909-024-05789-1

**Published:** 2024-07-24

**Authors:** Robin Walter, Leander Alt, Roman Hari, Michael Harris

**Affiliations:** 1 Institute of Primary Health Care (BIHAM), University of Bern, Mittelstrasse 43, Bern, CH-3012 Switzerland; 2 Dean’s office, Medical Faculty, University of Bern, Bern, Switzerland; 3 College of Medicine & Health, University of Exeter, Exeter, UK

**Keywords:** Undergraduate medical education, Ultrasound, Tutor characteristics, Evidence-based teaching, Qualitative research

## Abstract

**Purpose:**

This study was designed to elicit medical students’ opinions on the characteristics of a good ultrasound tutor. The results should help educators to create an optimal teaching environment and inform tutor training.

**Materials and methods:**

The qualitative study recruited 15 participants from a larger mixed-methods study of 64 medical students who underwent a basic course on abdominal ultrasound taught by faculty and near-peer tutors. During semi-structured interviews, they were asked which characteristics make a good ultrasound tutor. We used inductive thematic analysis to identify the most important categories.

**Results:**

Medical students identified teaching themes and subthemes relating to teaching skills (e.g., course structure, repetition, vocabulary, feedback, guidance of participants), tutors’ attitudes (e.g., atmosphere creation, empathy) and knowledge as the crucial components of being a good ultrasound tutor.

**Conclusions:**

While some of the themes that students identified are generic to medical education, others are specific to ultrasound teaching. Tutors can use our results to assess their own teaching. They should aim to address learning needs, optimise understanding, give adequate feedback, and create a non-threatening atmosphere with empathic interactions. Accounting for the ultrasound-specific setting they should possess the necessary knowledge, provide verbal guidance to their students, and distribute examination time wisely.

**Supplementary Information:**

The online version contains supplementary material available at 10.1186/s12909-024-05789-1.

## Background

In recent years, an increasing body of literature has not only investigated the optimal design of undergraduate teaching programmes, but also the characteristics of how tutors teach. This has most commonly been done for settings where tutors have a great impact due to intimate settings, such as clinical teaching in small groups [1–5].

High-achieving medical educators tend to have a distinctive set of characteristics. Sutkin et al. arranged their results from a qualitative analysis of the literature into three categories: Physician, Teacher and Human [4]. More recent studies have in essence only confirmed their findings, and sometimes added or removed certain subthemes. Adapted for clinical teaching, the Physician represents characteristics such as medical knowledge [2, 3, 6], clinical skills and enthusiasm for medicine [4]. The Teacher includes characteristics such as positive relationship with students, enthusiasm for teaching [2, 3, 5, 6], being accessible, giving frequent feedback and explanations and good management [2, 4, 6]. The Human category focuses on interpersonal aspects, such as creation of adequate course atmosphere [2–5], good communication [5, 6], use of role modeling [2], being an enthusiastic person and empathy [4].

More recently, a new setting has developed for undergraduate medical students: ultrasonography teaching now appears in many undergraduate curricula, many of which use near-peer tutors in addition to or to replace faculty tutors [7–9]. This creates a setting that is different to general clinical teaching, in that complex psychomotor skills are required to perform an ultrasound examination [1, 10–12]. The styles that tutors use for teaching ultrasound skills to undergraduates has so far received little attention in educational research. There is a particular need for this, as ultrasound teaching is a very intimate teaching setting coupled with technical skills learning.

In one study, Australian sonographers self-assessed their teaching approaches and identified coaching, verbalised demonstration, assessment of prior knowledge and feedback as frequently used teaching methods. Physical guidance was reported less often [12]. Another study observing ultrasound teaching by faculty and student tutors used the construct of cognitive apprenticeship [13], which seems a good fit to describe the steps within ultrasound teaching [14]: most teaching time was spent on coaching (observing and helping students), then articulation (asking stimulating questions) and finally modelling (demonstrating, giving explanation) [14].

However, students learning about ultrasound may have different perceptions as to what is important. This study was therefore designed to elicit medical students’ opinions on the characteristics of a good ultrasound tutor. The results should help educators to create an optimal teaching environment and help to them to design and optimise ultrasound tutor training.

## Methods

### Study design

This study used a qualitative research design, following a constructivist paradigm [15, 16]. Study participants were recruited from a larger mixed-methods study of 64 medical students who underwent a basic course on abdominal ultrasound taught by faculty tutors (FT) and near-peer tutors (NPT) [17].

### Study setting

The context of this study was a course focused on abdominal ultrasound, together with some teaching on thorax, neck, and basic musculoskeletal sonography. It consisted of five hours of e-learning, followed by 16 h of small-group hands-on teaching (2–4 learners per session, with learners taking it in turns to be the ultrasound models), concluding with a mandatory exam. For this study, four of the hands-on hours were taught by FTs and twelve by NPTs. The FTs were physicians who were experienced in the use (mean 11.5 years of experience) and teaching (mean 4.5 years) of ultrasound. The NPTs were medical students in years four to six who had 1–3 years of experience in teaching this course and had received training in ultrasound and didactic methods (basic abdominal ultrasound course plus three tutor-training-days with experts). All FTs and NPTs were familiar with the learning objectives as they regularly teach the content of this course. By including FTs and NPTs, a variety of tutors, could be experienced by the participating students.

### Participants and recruitment

The course participants were medical students at the University of Bern in their seventh semesters who participated in a blended learning ultrasound teaching programme from October 2020 to February 2021 [18]. These 64 students were selected at random from the 86 students who had stated an interest in participating in the teaching programme, out of a total of 220 seventh semester students.

After having completed the teaching programme and the mandatory final exam, all course participants had to fill in an online questionnaire to elicit participants’ demographic data and their opinions on the mixed setting of FT and NPT. The 15 interview participants were then selected based on their questionnaire answers, stratified for sex, group allocation and preference for faculty or near-peer teaching.

### Data collection

The 15 semi-structured interviews were carried out either online or in face-to-face meetings. All interviews were in Swiss German, which is not a written language. They were digitally recorded to allow for loss-reduced transcription to German by specially trained medical students. Interviews were performed throughout April and May 2021 and lasted 40 to 88 min. Portions of the interviews about the relative timing of FT- and NPT-led teaching are reported elsewhere [17].

LA performed three interviews and RW twelve. Both interviewers were University of Bern medical students who were NPTs senior to the participants. Out of 263 hands-lessons in this study LA taught 18, and RW 8 lessons. To reduce the risk of bias, the interviewers were trained on how to conduct the interviews by MH and RH, who are experienced qualitative researchers. Interviews followed an interview guide (see appendix) which was based on a review of the existing literature, discussion within the study team and answers from the online questionnaire. It was modified after two pilot interviews.

### Data analysis

We used inductive thematic analysis [19], an approach in which codes and themes are suggested by the data rather than by a theoretical framework. The phases of analysis included coding, followed by the identification and clustering of themes and subthemes, and the production of a descriptive thematic summary. To ensure consistent coding strategies, we developed a coding guide after comparing independent coding of two interviews by LA and RW and multiple meetings to look for inconsistencies and agreement. Once the coding was complete, more consensus meetings were held to discuss and agree the themes and subthemes.

We did not separate the participant’s statements in whether they concerned FT, NPT or both since our goal was to assess for important characteristics in general. Whether there is a difference between these two tutor groups was not the focus of this study.

### Ethical considerations

The ethics committee of Bern, Switzerland declared this study as not subject to the Human Research Act, and therefore not subject to the need for a comprehensive ethical appraisal (BASEC number Req-2020-01087). A reporting system was set up to enable review of unexpected pathological findings by experienced physicians within 24 h. All participants consented to the study and the plan for management of any unexpected ultrasound findings.

## Results

Of the 15 learners who were interviewed, 11 were female and 4 were male. No new themes emerged from the last two interviews, indicating data saturation. We identified three major themes: tutors’ teaching skills, which had both generic and ultrasound-specific components, and tutors’ attitudes and ultrasound-specific knowledge.

Themes and subthemes are described below, with quotations, translated into English by members of the study team, identified by a participant number.

### Teaching skills

Many of the necessary teaching skills mentioned by the participants are rather generic and are not limited to ultrasound teachings. The learners considered specific tutor teaching abilities to be crucial. Key skills for most participants included structuring the learning sequence carefully, aligning the content to the participants’ learning needs thereby enhancing the student-centred experience, and using learning objectives:

*PN25: “I liked when tutors checked all learning objectives together with the participants to fulfil each objective.”*

*PN31: “If tutors did not assess pre-course knowledge […]*,* they might be talking about the sides of the ultrasound image during the last course and all participants just think to themselves: ’Yes*,* we know about this.’”*.

Some participants felt their learning increased when tutors asked them relevant questions:

*PN52: “It is far easier to connect theoretical and practical knowledge*,* when tutors force participants to repeat theory with short inputs in between the hands-on parts. In this way teaching becomes more sustainable.”*

Most participants agreed, that tutors needed to be able to adapt their explanations to the learners’ level and regularly check for understanding so that problems could be recognised and adequately addressed by the tutor:

*PN32: “Especially in the beginning*,* it is crucial for tutors to be able to explain simply and good since there is no knowledge present yet.”*

*PN63: “For some tutors it was difficult to split their large knowledge in little handy parts for us to understand. […] [Tutors] should be able to explain well and alternatively use drawings*,* models*,* or other aids to explain.”*

*PN33: “Tutors should directly correct errors once they see them occurring […] since all participants only have very little experience and automatically conclude everything was done correctly if no correcting comments follow.”*

Finally, meaningful and timely feedback was appreciated by most participants:

*PN36: “Tutors don’t need an incredible amount of knowledge*,* such as clinical knowledge*,* but should rather know very well where to find the different organs and should know how to correct. [Learners] need to know when something was performed right or wrong*,* so they know which parts to keep and which to discard.”*

*PN11: “What is cool is when you as participants are performing the examination and the tutor is immediately handing you propositions on what and how to improve*,* and I prefer it formulated in a positive way“*.

The following findings seem to be more specific to ultrasound courses.

In our setting, where learners carried out ultrasound tasks while observed and supported by their tutors, most learners preferred tutors to give verbal guidance rather than to take over the ultrasound probe themselves. This allowed them to understand how the tutors were thinking. If tutors needed to take over the probe, participants preferred to keep holding onto it as well. Some learners also stated they liked tutors to demonstrate the ultrasound examination sequence:

*PN31: “I think it is very important for participants to do the hands-on part*,* not the tutors.”*

*PN08: “When the tutor takes over the probe without you holding on to it*,* then you don’t have a feeling for the changes. This improves if the tutor takes over with you together*,* so you still get a feeling for the performed action.”*

*PN36: “Especially when it was the first time performing [ultrasound]*,* I liked [tutors] to first demonstrate the examination themselves.”*

Some participants thought, tutors could enhance the hands-on experience through adequate structuring which would allow for hands-on time to be evenly distributed among participants:

*PN01: “There is little time*,* and it has to be used effectively. […] If there is no structure in the sequence*,* [time for] hands-on ultrasound teaching is not evenly distributed between participants*,* which is a disadvantage.”*

Participants liked when tutors explained their reasons for how they moved the probe:

PN08: “Accompanying with words would have helped me more to clarify the necessary considerations.”

*PN60: “I think tutors should be able to explain why they do the movements when showing [them].”*

### Tutors’ attitudes

Most of our participants valued interpersonal skills in terms of a tutor’s attitude. A key element of this was the ability to create a comfortable atmosphere, enabling learners to ask the most basic questions:

*PN11: “It makes a huge difference when one is in a good mood [as learner]*,* learning effects are far greater. Even if you arrive in good mood*,* this can be increased by a [tutor] that makes participants laugh. Participants will feel safer to use trial-and-error tactics. Also*,* participants dare more to ask questions.”*

*PN33: “Course quality depended on who gave the course*,* and I mostly liked when they gave me the feeling that even the most basic question is allowed. In this way I can continue to thrive.”*

Tutor expectations that were too high could have the opposite effect, and this could affect learning:

*PN52: “When a tutor’s expectations are too high*,* participants won’t dare to ask any questions and the course then does not really serve a purpose.”*

Most learners liked tutors who were patient, friendly, with outgoing behaviour, enthusiasm, and a desire to teach:

PN64: “[A good tutor] is one that is responsive to participants and is friendly.”

*PN52: “[A good ultrasound tutor] should not be timid or reserved*,* so that [learners] would have to ask explicitly for every little bit of information.”*

Tutors being empathetic was also seen as a good quality:

*PN32: “A helpful ultrasound tutor […] would be able to understand and answer my questions in a helpful way as if [he or she] was able to put themselves in my situation.”*

### Tutors’ knowledge

Learners stated that the tutors should have the abilities and knowledge necessary for ultrasound teaching. This related to knowledge about the course content, as well as that needed to answer further questions, both of which were considered to be essential for professionalism in an ultrasound course:

*PN25: “When the goal is to impart knowledge to participants*,* tutors need to have the knowledge ready themselves and explain in ways that participants understand.”*

PN38: “If tutors can answer to additional questions, their professionalism increases.”

## Discussion

### Principal findings

Medical students participating in a course on basic ultrasound identified teaching skills, tutors’ attitudes and knowledge as the key ingredients to being a good ultrasound tutor: they wanted general teaching skills to include the circumstances and structuring of the course, asking stimulating questions, adaption of explanations to participants’ levels, and giving relevant feedback; and also ultrasound specific teaching skills, such as a preference for verbal guidance, accompanying explanations to every movement, and evenly distributed hands-on time. They liked tutors’ attitudes to focus on atmosphere creation, empathy, as well as adequate and outgoing behaviour; and they felt that tutors need both the necessary background knowledge and the ability to answer any additional questions that arose.

### Comparison with existing literature

Our findings confirm those of other studies, in which learners in a variety clinical teaching settings expect knowledge of the subject, enthusiasm, and creation of a good course atmosphere [2–6]. Also, good course management has previously been identified as being a key characteristic of a good clinical teacher [2, 4, 6]. Our finding that teaching skills, and topic-related knowledge are important reflects those of other investigators [2–6].

Tutors’ attitudes as found in our study reflects the theme of the “Human” found by Sutkin et al. [4], since this includes good communication, with approachable and empathetic behaviour. We did not, however, find some of the elements described by Sutkin et al., such as the need for role modelling or respect; this might be due to the more limited and very intimate setting of ultrasound teaching, because it forces tutors and students to focus on one specific topic and pay more attention to each other’s private sphere.

The teaching skills that are specific for ultrasound teaching may exist due to the complexity of ultrasound and its technical aspects [10]. The participating students were rarely confronted with technical skills before, since they just started their clinical training. This might be why they liked to have as much hands-on time as possible on one hand while not lacking close, mostly verbal, guidance by the tutor on the other hand. While there is no ultrasound-specific literature reflecting this, there are generic instructional models for technical skills teaching that focus on verbal guidance [11, 20]. Models on clinical teaching also promote frequent verbalisation of thoughts and processes [21].

Students wanted to know the reasoning behind any error corrections, so that they could adapt their mental processes, which also fits well to existing recommendations for clinical teaching and problem-based learnings [21, 22]. Administrative skills concerning course organisation, such as participants wanting examination time to be distributed evenly, did not arise in other studies so far, as was also stated by Wondwossen Fantaye et al. [5].

### Strengths and limitations

This is the first study to focus on medical students’ views on the characteristics of a good ultrasound tutor. The research team consisted of both medical students and medical faculty. The team carefully developed and piloted the interview questions. Each student had experienced teaching by multiple tutors, with a mix of faculty and near-peer tutors. This gave a broad spectrum of teaching, which allowed participants to experience a complex and more complete picture of tutor abilities. We achieved data saturation.

This study’s limitation was the narrow recruitment strategy, as all participating students were in their fourth year of medical studies. This was a pragmatic decision, so that we could interview students who had both experienced near-peer and faculty teaching. Still, this narrow recruitment might limit generalisability since more interviews with more advanced students may have given us additional insights. Social desirability bias was possible, as participants may have answered questions in a way that they thought would be viewed favourably by the researchers.

### Implications for practice and research

Our findings provide the basis for guidance on how ultrasound tutors should approach practical, hands-on ultrasound training, with both generic and ultrasound-specific recommendations (Table 1).

**Table 1:** Themes, subthemes and their implications

Theme	Subthemes	Implications Ultrasound tutors should…
Teaching skills - generic	Structure Learning needs Learning objectives	Tutors should find out what the participants’ learning needs are and explain how the teaching sequence is structured around these and the learning objectives.
	Questions Level adaption Checking for understanding	When asking questions or explaining, tutors should use a language that is easily understood by learners, and regularly assess the learners’ understanding.
	Feedback	Timely feedback helps students learn from their mistakes immediately and deepens their understanding.
Teaching skills - ultrasound-specific	Verbal guidance Explanations for every movement	When learners have difficulties in achieving the desired image, tutors should describe how to manipulate the probe to improve the image and explain why these particular techniques are needed. If tutors still need to take over the probe, they should do this while simultaneously guiding their learner’s hand.
	Examination time during teaching	Tutors should ensure regular rotations of the student examiner, so that all learners have a similar amount of practical training time.
Tutors’ attitudes	Atmosphere Expectations	Tutors should be non-threatening but show enthusiasm and humour. They should not have an unreasonably high expectation of what the learners can do.
	Empathy	Tutors should try to understand their learners’ perspectives to better understand their problems and needs.
Tutors’ knowledge	Ultrasound-specific knowledge	Tutors need to be prepared for any additional questions that they may be asked.

Participants of an ultrasound course do want teachers that know their topic well, but they wish for much more than just that. All the data we gathered shows a need for didactical training in addition to knowledge and performance training. In this didactical training, tutors-to-be should be provided with feedback on how they structure a course including efficient and equilibrated examination time, the clarity of their instructions, how often and how well they give feedback to single participants and how they can improve the atmosphere of a course. Additionally, tutors should be trained in verbal guidance of a student performing an ultrasound exam, for example by guiding a blinded examiner to create a typical ultrasound image using unambiguous language. Such exercises can prove the value of a standardised vocabulary and the power of verbal guidance to new tutors.

Further studies should consider whether our findings are relevant to advanced and postgraduate ultrasound courses and should investigate whether tutors’ perspectives give additional insights. Further quantitative research is necessary to investigate the differences between FT and NPT concerning characteristics of good tutors to close another knowledge gap.

## Conclusion

Medical students who were learning practical ultrasound skills gave their perceptions on the characteristics of a good ultrasound tutor. While some of the themes that they identified are generic to medical education, others are specific to ultrasound teaching. Tutors can use our results to assess their own teaching. They should aim to address learning needs, optimise understanding, give adequate feedback, and create a non-threatening atmosphere with empathic interactions. Accounting for the ultrasound-specific setting they should possess the necessary knowledge, provide verbal guidance to their students, and distribute examination time wisely. We highly encourage medical educators and individual tutors to use our findings to improve their teaching or their teachers’ training.

Michael Harris, Prof., MD, MB BS, FRCGP, MMEd, is a British General Practitioner and a Research Associate at the Institute of Primary Health Care (BIHAM), University of Bern, Bern, Switzerland and at the College of Medicine & Health, University of Exeter, Exeter, UK.

### Electronic supplementary material

Below is the link to the electronic supplementary material.

Supplementary Material 1

## Data Availability

The datasets used and analysed during the current study are available from the corresponding author on reasonable request.

## Abbreviations

FT: faculty tutors
NPT: near-peer tutors
N: number
