# Supplementary material for: What do medical students think are characteristics of a good ultrasound tutor? A qualitative study
Source: BMC Med Educ. 2024 Jul 24;24:796. doi: 10.1186/s12909-024-05789-1 (PMC11270876; doi:10.1186/s12909-024-05789-1)
Supplement: Supplementary file 1 — Supplementary Material 1 [file 12909_2024_5789_MOESM1_ESM.docx]

# Appendix

## Interview proforma

Only questions relevant to this study are being displayed.

| Gender: | Participant Number: |
| --- | --- |

| Open ended Question | Prompts (use if not covered by initial response) |
| --- | --- |
| General demographics and background info | |
| Would you be happy to tell me your age? |  |
| What other ultrasound experience do you have apart from this blended learning?  *Follow up if not explained:* When did you gather these ultrasound experiences? | WP-abdomen, WP-neck, E-Fast, POCUS, during internship in the hospital, at my GP-tutor |
| What teaching experience did you gather yourself? | Peer-tutor (in medical courses), giving extra tuition, teaching a class |
| What other courses did you already experience, who had both near-peer and faculty-led tutors? | practical medical courses |
| Differences of faculty-led and near-peer tutors  short transition (all about ultrasonography tutors) | |
| What are important characteristics of a good ultrasound tutor for you?  *Follow up if not explained:* What does this characteristic mean for you?  Make short notes of answers | - General characteristics for a tutor - Ultrasound specific characteristics |
| Out of those, what is the most important single characteristic of a good ultrasound tutor for you?  *Follow up if not explained:* What does this characteristic mean for you? |  |
| What are bad characteristics of ultrasound tutor for you?  *Follow up if not explained:* What does this characteristic mean for you?  Make short notes of answers | - General characteristics for a tutor - Ultrasound specific characteristics |
| Look at notes:  What differences did you remark between faculty and near-peer ultrasound tutors regarding *”noted answer”*?  *Follow up if not explained:* How did you experience these differences during your ultrasound lessons? | - competency in the field, - ability to give advice on what you should do to improve the image on your own, - ability to correct the position of the ultrasound device in an adequate amount of time, - giving constructive feedback, - explaining complicated topics in an understandable way, - ability to give satisfying answers, - ability to give clinical inputs, - tutor not paying attention to the class - tutor embarrassing a participant |
| How do you compare the atmosphere in the faculty-led and near-peer led lessons? | - Relaxed or high pressure - Feeling allowed to take risks and do mistakes or expectations to already know everything - Feeling accepted or being embarrassed - Motivating or demotivating |
| [Question about specific experiences with faculty tutors] |  |
| [Question about specific experiences with near-peer tutors] |  |
| Optimal combination of faculty-led and near-peer teaching | |
| [Various questions about optimal timing of near-peer and faculty teaching, preferences and challenging learning content] |  |
| Optimal organisation of the lessons | |
| [Various questions about the organisation of ultrasound classes] |  |
